# Type I fimbriae subunit *fimA* enhances *Escherichia coli* biofilm formation but affects L-threonine carbon distribution

**DOI:** 10.3389/fbioe.2022.904636

**Published:** 2022-10-21

**Authors:** Qingguo Liu, Jiaqing Zhu, Na Liu, Wenjun Sun, Bin Yu, Huanqing Niu, Dong Liu, Pingkai Ouyang, Hanjie Ying, Yong Chen, Gulin Zhao, Tianpeng Chen

**Affiliations:** ^1^ State Key Laboratory of Materials-Oriented Chemical Engineering, College of Biotechnology and Pharmaceutical Engineering, Nanjing Tech University, Nanjing, China; ^2^ Nanjing Hi-Tech Biological Technology Research Institute Co., Ltd., Nanjing, China

**Keywords:** *Escherichia coli*, *fimA* gene, biofilm, L-threonine carbon distribution, transcriptomic analysis

## Abstract

The biofilm (BF) provides favorable growth conditions to cells, which has been exploited in the field of industrial biotechnology. Based on our previous research works on type I fimbriae for the biosynthesis of L-threonine (LT) in *Escherichia coli*, in this study, a *fimA*-overexpressing strain was engineered, which improved BF formation under industrial fermentation conditions. The morphological observation and characterization of BF formation were conducted to verify the function of the subunit FimA. However, it was not suitable for repeated-batch immobilized fermentation as the LT titer was not elevated significantly. The underlying molecular mechanisms of BF formation and the LT carbon flux were explored by transcriptomic analysis. The results showed that *fimA* regulated *E. coli* BF formation but affected LT carbon distribution. This study will stimulate thoughts about how the fimbriae gene regulated biofilms and amino acid excretion and will bring some consideration and provide a reference for the development of BF-based biomanufacturing processes in *E. coli*.

## Introduction

L-threonine (LT) is an essential amino acid with a variety of functions in pharmaceutical, chemical, and food industries, as well as feed additives (Leuchtenberger et al., 2005). Thus, its demand is sharply increasing. At present, microbial fermentation is generally used for LT synthesis in the industry due to its low raw material cost, high fermentation yield, and easy large-scale production. Moreover, *Escherichia coli* (*E. coli*) has become the best candidate strain in industrial production with the characteristics of easy culture and a clear genetic background (Lee et al., 2006; Dong et al., 2011). However, the preparation of the seed culture was required every time in free-cell batch LT fermentation, which led to the unavailability for the continuous catalytic process (Beloin et al., 2004; Rajkumar et al., 2013). In addition, the increasing products and salt concentrations of *E. coli* might affect the membrane osmotic pressure for cell tolerance, and then, the imbalance of cell homeostasis could result in a low reaction intensity for LT production.

Immobilized fermentation based on a continuous catalytic process is one of the most important ways to realize the continuous production of chemicals, which has gained worldwide attention. Hence, establishing a new immobilized fermentation system can achieve continuous and efficient production of LT in *E. coli*. The biofilm (BF) is a complex cell community closely related to biotic or abiotic surfaces (Sauer, 2003). Cells can employ these structures to withstand shear forces and obtain nutrients. *E. coli* covered by BFs can tolerate extreme conditions, including high cell density, hypoxia restriction, and high osmotic pressure, which are essential for the continuous fermentation process.

BF-based immobilized fermentation is considered a solution instead of free-cell fermentation, owing to its unique advantages (e.g., BF matrix protection, repeated cells, and improve metabolic activities) compared to free-cell fermentation (Zhao et al., 2015). The microbial BFs, such as *Aspergillus niger*, *Saccharomyces cerevisiae*, *Clostridium acetobutylicum,* and *E. coli*, have been effectively used for immobilized fermentation (Chen et al., 2013; Liu et al., 2013; Yu et al., 2018; Chen et al., 2019).

Type I fimbriae, about 1–2 μm in length and about 6.9 nm in width, are one of the most critical structures for BF biosynthesis in prokaryotic bacteria, including *E. coli*, which contain a large number of fimbrial gene clusters belonging to the chaperone/usher assembly class (Tripathi et al., 2013). Each operon encodes type I fimbriae structural subunits that are assembled into a fimbrial filament on the cell surface by a periplasmic chaperone and outer membrane usher protein (Hung and Hultgren, 1998). Previous research has confirmed that the overexpression of *fimH* could improve the efficiency of LT production by enhancement of BF formation and immobilization intensity (Chen et al., 2019). Moreover, it has been reported that FimA is the main structural unit of type I fimbriae, which consists of 500–3000 FimA proteins, accounting for 95%. Meanwhile, fimbriated bacteria can express 4200 fimbrial filaments on their surface, which plays a key role in the fimbriae assembly and BF formation (Berne et al., 2015). Thus, upon expression of a fimbrial gene cluster, the respective major structural subunit could become one of the most abundant proteins in the bacterial cell.

In the present study, a *fimA*-overexpressing *E. coli* strain was constructed for enhancing BF formation. A BF-based fermentation system was developed with polyurethane as a carrier for BF enrichment. After adhesion to the carrier surfaces, the cells formed a mass of BFs to withstand high shear force. The morphological observation was performed for the characterization analysis of BFs. In addition, the effects of *fimA* disturbance on the microbial physiological process of *E. coli* were explored, in combination with the detection of LT yield. Subsequently, transcriptomic analysis was conducted to explore to the role of *fimA* disturbance in cell physiological metabolism.

## Material and methods

### Plasmids and strains

*E. coli* W1688 (CCTCC M2015233), an LT-producing strain, was derived from *E. coli* MG1655 by introducing mutations and molecular modifications of the LT metabolic pathway, and it could form BFs. All detailed characteristics of plasmids and strains in this study are presented in Table 1. Amplification of the *fimA* gene was performed using the *E. coli* W1688 genomic DNA. The plasmid pET28a and the *fimA* gene were ligated with the restriction enzymes *Xba* I and *Nco* I using the ClonExpress II One Step Cloning Kit (Vazyme, Nanjing, China). Red homologous recombination was used in the *fimA* deletion strain of *E. coli* W1688 (Datsenko and Wanner, 2000; Madyagol et al., 2011). Briefly, the gene of the kanamycin resistance sequence and homologous regions (about 50 bp) were combined by PCR. Subsequently, the One Step Cloning Kit (Vazyme, Nanjing, China) was used in the cloning of the plasmid pKD4 and the resistance fragment. The knockout of the gene *fimA* was performed by electroporation (Bio-Rad, United States) with 2.0 kV, 25 mF, and a 200 Ω pulse with the recombinant plasmid transforming into the *E. coli* W1688 strain. The specific operation processes were adopted from the study by Chen et al. (2019).

**TABLE 1:** Strains and plasmids used in this study.

Strain or plasmid	Relevant characteristic	Sources
Strains		
*E. coli* W1688^a^	L-threonine-producing strain	Prof. Sheng Yang
*E. coli* W1688-fimA*	*E. coli* W1688-harboring plasmid pET28a-*fimA*	This study
*E. coli* W1688-ΔfimA	*E. coli* W1688 with the deletion of *fimA*	This study
*E. coli* W1688-pKD46	*E. coli* W1688-harboring plasmid pKD46	This study
Plasmids		
pET28a	Kan resistance	Datsenko and Wanner (2000)
pET28a-*fimA*	pET28a-containing *fimA*	This study
pKD46	Amp resistance	Datsenko and Wanner (2000)
pKD4	Kan resistance	Datsenko and Wanner (2000)

^a^ A gift from Prof. Sheng Yang (Institute of Plant Physiology and Ecology, CAS, Shanghai, China).

### Media and growth conditions

The wild-type and recombinant strains of *E. coli* were cultured in an LB medium containing NaCl (10 g/L), tryptone (10 g/L), and yeast extract (5 g/L). The fermentation medium contained glucose (30 g/L), (NH_4_)_2_SO_4_ (20 g/L), CaCO_3_ (15 g/L), yeast extract (2 g/L), KH_2_PO_4_ (1 g/L), MgSO_4_·7H_2_O (0.8 g/L), MnSO_4_·5H_2_O (0.2 g/L), and FeSO_4_·7H_2_O (0.2 g/L). The initial pH was adjusted to 7.2 using acetic acid. All culture media were sterilized at 115°C for 20 min, and 0.5 mM isopropyl-β-D-thiogalactopyranoside (IPTG) was added when necessary. The shake flask fermentation was operated at 37°C and 200–220 rpm.

### Immobilized and free-cell fermentation

Briefly, 5% seed culture with final OD_600_ 0.05 was inoculated in free-cell fermentation with flasks. Sampling was performed, followed by centrifugation (8000 rpm, 5 min, 4°C), and the supernatants were subjected to the quantitative analysis of LT and residual glucose. Immobilized fermentation was conducted with the carrier (30 g/L), according to the aforementioned conditions. After the first batch, 10% of the residual broth with the carrier covered by BFs was added into the fresh culture medium, followed by the second batch of culture. The subsequent operations were the same as mentioned earlier.

### Analytical measurements

The concentrations of LT were determined by HPLC (high-performance liquid chromatography) with a UV detector at 338 nm, according to its maximum UV absorption wavelength, 1 ml/min, and 36°C. The mobile phase consisted of 80% acetonitrile and 0.1 M sodium acetate. The concentrations of glucose were detected using an RI detector with the Aminex HPX-87H column at 1 ml/min and 55°C. The mobile phase consisted of 5 mM H_2_SO_4_.

Total biomass staining was performed for the semi-quantitative analysis of BFs using the basic dye crystal violet (Li et al., 2003). After 12-h cultivation at 37°C, the strains were diluted (1:2000) and cultured again in a 96-well plate (200 μl/well) at 37°C. PBS (1%) was used to wash the wells twice for removing free cells. The BFs were fixed with methanol at 4°C for 15 min. Subsequently, the BFs were stained with crystal violet (1%) for 15 min, and PBS was used to wash the wells repeatedly 3–4 times. Then, 200 μl of acetic acid (33%) was added to dissolve crystal violet. Finally, the absorbance was measured at 570 nm using a Thermo Scientific microplate reader (Multiskan SkyHigh, Thermo Fisher Scientific, United States).

After 30-h cultivation, the coverslip was gently removed from the fermentation broth for the observation of BFs through microscopy. The BFs were fixed in paraformaldehyde (4%) and dehydrated using a vacuum freeze dryer. Transmission electron microscopy (TEM) (HT7820, Hitachi, Japan), TM3000 electron microscopy (Hitachi, Japan), and scanning electron microscopy (SEM) (SEM 4800, Hitachi, Japan) were carried out.

The adhesion properties of bacteria and distribution of cells in the BFs were examined using a fluorescence microscope. Briefly, 4% paraformaldehyde was used for BF fixing as described previously. Then, the fixed BFs were stained with 0.2 μg/ml 4′,6-diamidino-2-phenylindole (DAPI) for 30 min.

### Transcriptomic analysis

The pretreatment steps of the three strains before transcriptomic analysis were adopted from the study by Chen et al. (2019). Frasergen Inc. (http://www.frasergen.com/) provided sequencing platforms and technical assistance in data analyses. Briefly, the samples of immobilized fermentation were centrifuged (8000 rpm, 5 min, 4°C) to remove the liquid supernatant. Three biological replicates were applied for parallel analysis. The differential expression level of each gene was assessed according to the absolute value of Log_2_Ratio ≥ 1 and a false discovery rate (FDR) of ≤ 0.05.

### Real-time quantitative PCR assays

After harvesting *E. coli* recombinant strains and wild-type strains at the exponential phase, the RNAprep Pure Cell/bacteria Kit was used to isolate total RNA, and RNase-Free DNase was used to digest the residual DNA. cDNA was prepared for RT-qPCR after reverse transcription. The primer pairs were designed by Primer Express software with avoidance of choosing hairpin structures (Table 2). RT-qPCR assays were conducted on a StepOnePlus Real-Time PCR System using the SYBR Green PCR Master Mix. The relative expression levels of target genes were measured using the 2^-ΔΔCt^ method. One negative control and three technical replicates were used for each sample.

**TABLE 2:** Genes and primers used for quantitative real-time PCR.

Gene	Forward primer sequence	Reverse primer sequence
*fimA*	CCT​GAA​TAA​CGG​AAC​CAA​TAC​CA	GCCCCGGTTGCAAAATAA
*flhD*	GAC​AAC​GTT​AGC​GGC​ACT​GA	TTG​ATT​GGT​TTC​TGC​CAG​CTT
*flhC*	TCA​GGA​AGC​GCG​GGA​TAT​T	GAGCGCCCAGGGTGATC
*fliC*	CGT​ATT​AAC​AGC​GCG​AAG​GAT	GAG​GTG​AAA​CGG​TTA​GCA​ATC​G
*fliD*	GCGCGGCTAACTCATCGT	GCC​TGC​TTT​TGC​GTT​GTT​G
*fliG*	CGA​TGA​GCA​TCC​GCA​AAT​T	TGGGCGCGCTTCAGA
*flgG*	GGG​CAC​AGT​CTT​CCG​AAC​A	CCC​CGT​GCC​GAT​TTG​TAA​T
*thrB*	CGA​GCT​GGA​AGG​CCG​TAT​C	AAC​ACG​GTG​CCA​CGT​TGT​C
*rhtA*	TCG​TCG​CCC​GGT​AGA​TTT​C	GCA​GGA​ACC​ACA​GAC​CAA​GAA
*purA*	CCG​ACC​GAA​CTG​TTT​GAT​GA	GAA​TTC​GTT​ACC​CTG​CTT​GCA
*cysE*	CCGCTGACCCGGAAATG	TAC​GCA​CCG​CCT​GAA​TAT​CA
*sdhD*	GCAACGCCTCCGCATTAG	GCG​CGA​ACG​AGG​ATG​AAA​T
*argG*	CTG​ATA​CCC​GCG​AGA​AAC​TTT​T	CGGCAGAGGAGGAAAGCA
*ackA*	GTA​TTT​GAC​ACC​GCG​TTC​CA	GGC​AGG​GCG​TAG​AGG​TAA​GA
*acs*	TGGGCGAGCCAATTAACC	CTC​GTT​GCC​GAT​TTT​TTT​CC
*16s RNA*	TCG​GGA​ACC​GTG​AGA​CAG​G	CCG​CTG​GCA​ACA​AAG​GAT​AAG

The annealing temperature of PCR in the StepOnePlus Real-Time PCR System was always 60°C.

## Results and discussion

### Biofilm formation in the recombinant strains

**FIGURE 1:**
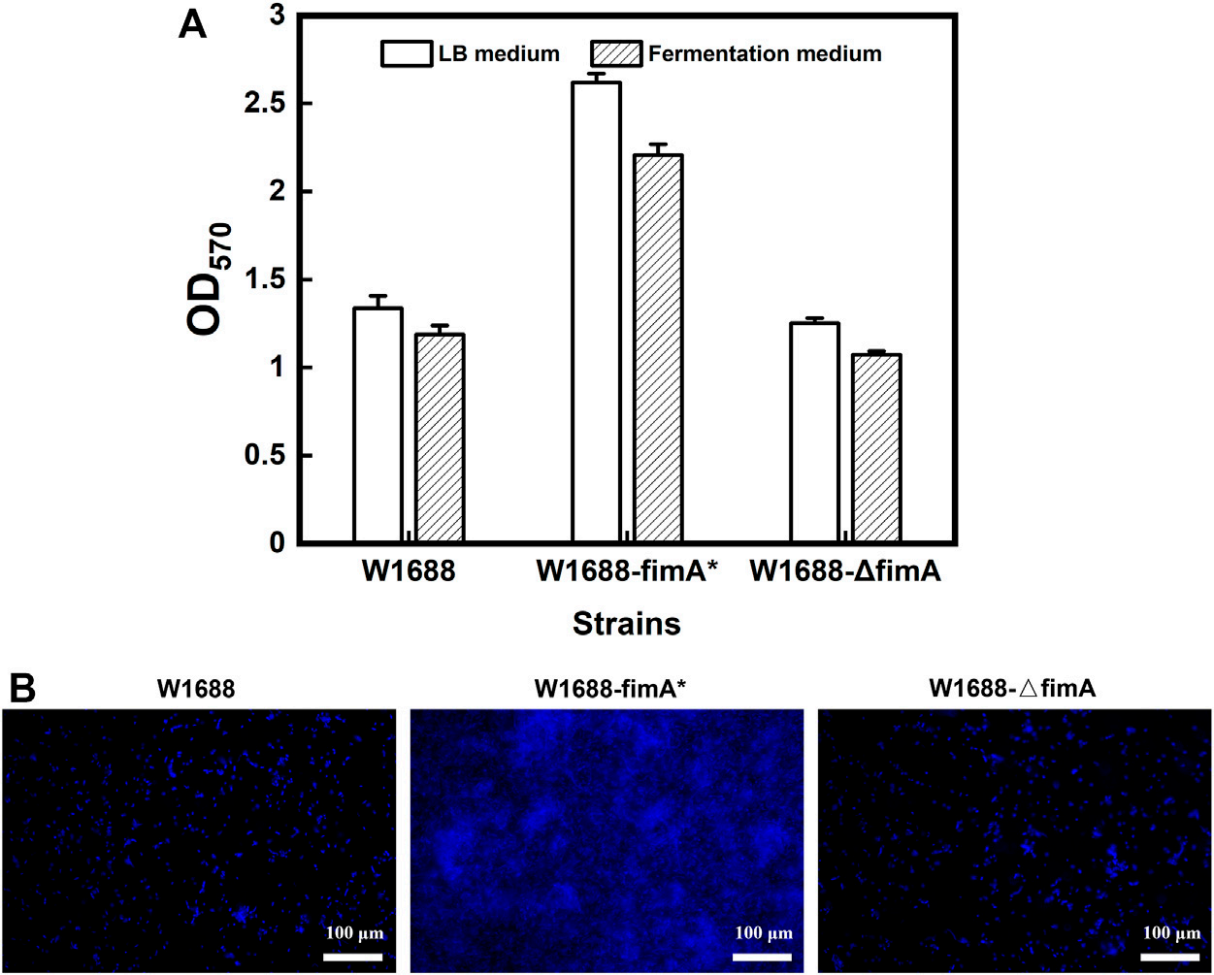
Semi-quantitative analysis of BF formation in the three strains. **(A)** Crystal violet staining in the two media. **(B)** Fluorescence microscopy of the BFs stained by DAPI.

After spread plate cultivation, negative control and false positive test were carried out simultaneously. Then, 549 bp of the whole *fimA* fragment was deleted by the replacement of kanamycin resistance with the results of PCR and sequencing verification, which was named *E. coli* W1688-ΔfimA. For the *fimA* overexpression strain, *E. coli* W1688-fimA* was also successfully constructed. The different abilities of BF formation were revealed by the 96-well plate experiments. The crystal violet staining OD_570_ of W1688-fimA* in the LB medium was remarkably elevated by 96% compared to that of the wild-type strain (1.34 versus 2.62, respectively), which could be benefited by *fimA* overexpression (Figure 1A). By contrast, the knockout of *fimA* gene markedly attenuated BF formation in the W1688-ΔfimA strain compared to the wild-type strain (1.34 versus 1.25, respectively). Consistent results were also found in the fermentation medium. Actually, after the construction was completed, the difference between the vector control alone with pET28a and the wild-type strain was less than 5% in the aspect of the microbial growth, biofilm formation, and amino acid production. Therefore, these data were omitted. In addition, the fluorescent dye DAPI was used to observe the living cells, which preliminarily verified the effect of *fimA* on BF formation.

**FIGURE 2:**
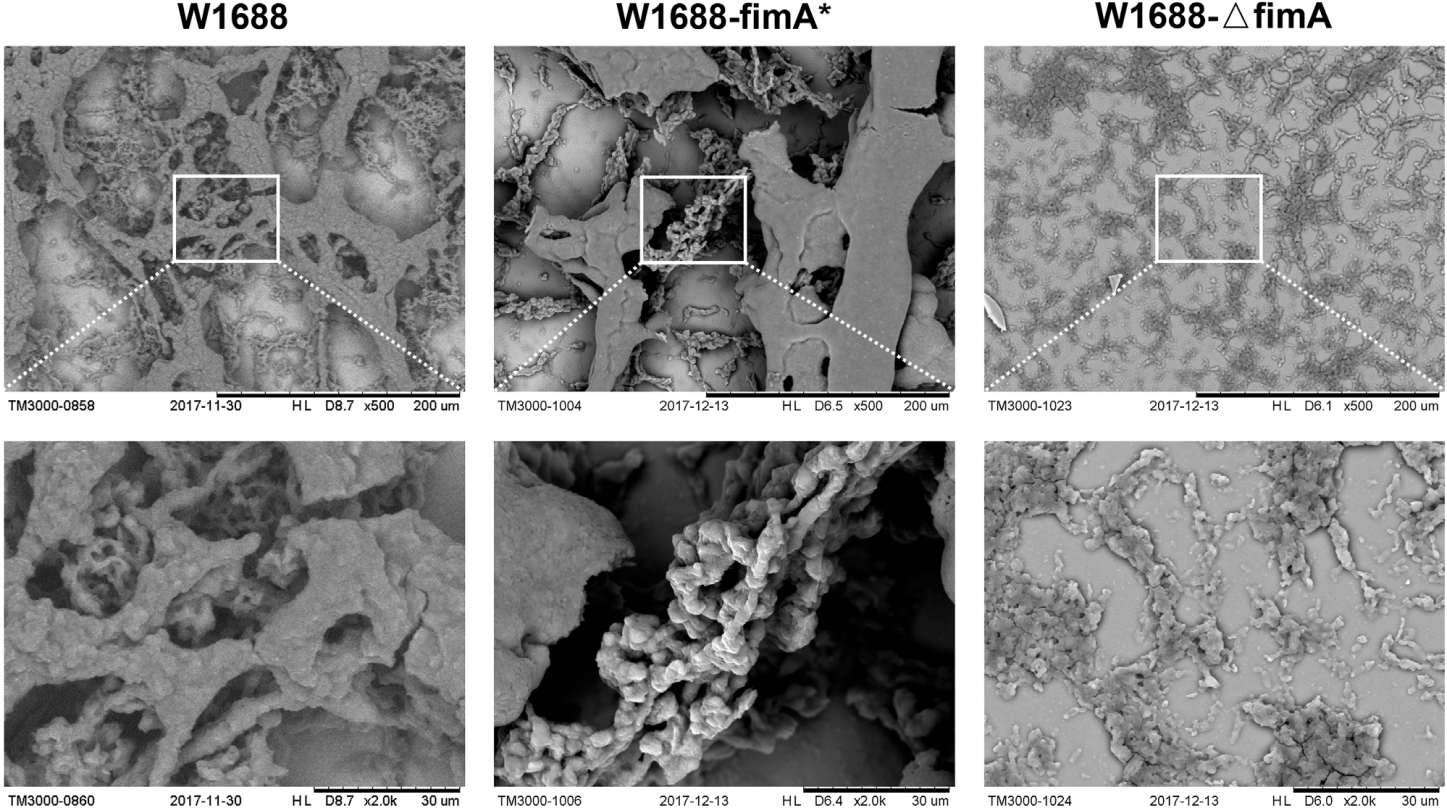
TM3000 electron microscopy of cell adhesion and BF formation at various magnification levels. First column: W1688; second column: W1688-fimA*; third column: W1688-△fimA. Cell adhesion and BF formation were noticeable in *E. coli* W1688-fimA*. Cells were encased in BFs by extracellular secretions with the form of aggregation with each other. BF formation was decreased and presented as a sparse distribution in *E. coli* W1688-ΔfimA.

**FIGURE 3:**
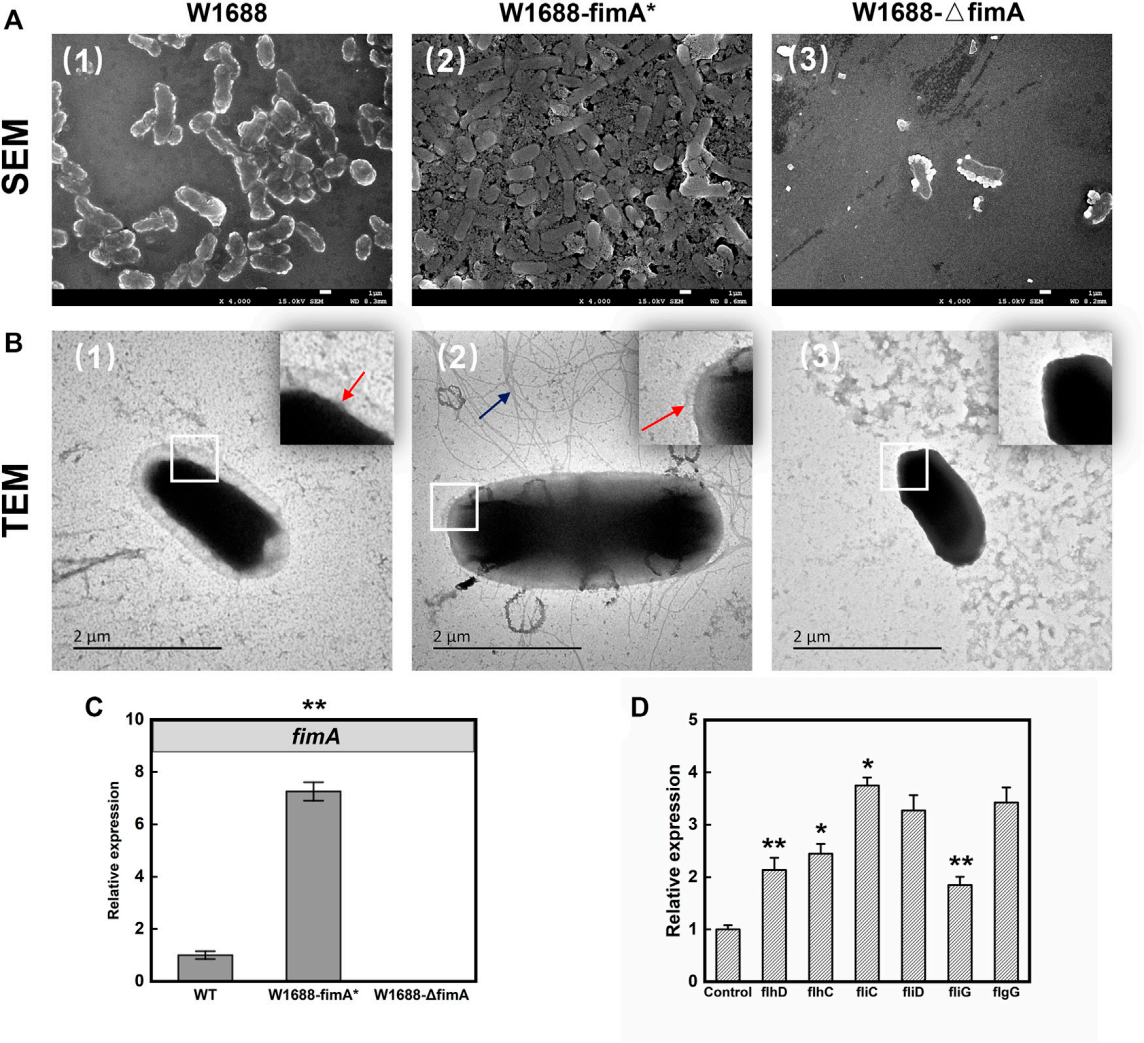
SEM **(A)** and TEM **(B)** images of BF formation and cell adhesion. First row: SEM; second row: TEM. First column: W1688; second column: W1688-fimA*; third column: W1688-△fimA. **(C)** RT-qPCR validation of the *fimA* expression. **(D)** RT-qPCR validation of flagellar gene expression in WT and the fimA* strain. Mean ± SD (*n* = 3). Two-tailed *t*-test. ***p < 0.001, **p < 0.01, and *p < 0.05. The results of SEM for the observation of BF formation were similar to crystal violet staining of the BF. Type I fimbriae were tiny on the cell surface (Figure 3B1, the red arrow). The absence of fimbriae with smooth appearance was observed in W1688-△fimA (Figure 3B3). An increase was observed in the extracellular surface fimbriae in W1688-fimA* (Figure 3B2, the red arrow), and the accumulation of the flagellum assembly with the filamentous substance attached to bacteria was observed in W1688-fimA*, which intertwined cells with each other (Figure 3B2, the blue arrow).

TM3000 electron microscopy indicated that cell adhesion and BF formation were more noticeable in *E. coli* W1688-fimA* at different magnifications than those in the wild-type strain (Figure 2). Cells were encased in BFs by extracellular secretions, which were in the form of aggregation with each other. On the contrary, BF formation was obviously decreased and presented as a sparse bacterial distribution in *E. coli* W1688-ΔfimA. In addition, SEM was also used for the observation of BF formation (Figure 3A). The physiological function of *fimA*, as the subunit of type I fimbriae, was investigated. Based on the results of TEM, tiny type I fimbriae were seen in the wild-type strain (Figure 3B1). As expected, the deletion of the *fimA* gene resulted in the absence of fimbriae with a smooth appearance (Figure 3B3). This may be due to the fact that FimA is the main structural unit in the fimbrial assembly, which account for 95%. In contrast, the overexpression of *fimA* not only contributed to an increase in fimbriae which assembled on the cell surface evenly but also led to the accumulation of flagella with the filamentous substance attaching to bacteria, which intertwined cells with each other (Figure 3B2).

RT-PCR test results showed the relative expression of fimA in the fimA* strain was seven times more than that in the wild-type strain (Figure 3C), which indicated that due to the high efficiency of the T7 promoter, the *fimA* gene could still be activated efficiently when IPTG was added at the beginning of the batch fermentation. To investigate the increased flagellum in the fimA* strain, RT-PCR was used to detect the expression of some representative genes in the flagella. The results showed that the transcriptional activators of the flagellar regulon (FlhDC), flagellar filament structure protein (FliC), flagellar filament capping protein (FliD), flagellar motor switch protein (FliG), and flagellar basal-body rod protein (FlgG) significantly increased with varying degrees (Figure 3D), suggesting that the overexpression of *fimA* gene may enhance the assembly of the flagellum structure. Here, we describe the phenomenon that overexpression of *fimA* could result in an increase in flagella. The expression of flagella could represent two meanings: one was the expression of flagellum composition and structure, which led to the increase in the flagellum assembly. The other one was the expression of flagellum-mediated motility with energy expenditure, which is represented by the flagellar motor gene *motA/B*. The overexpression of the *fimA* gene might enhance the assembly of the flagellum structure, and RT-PCR results for relative expression of *motA/B* had not shown a significant change (data not shown), which indicated *fimA* overexpression might be not associated with the activation of bacterial motility directly. It was similar that Lane et al. (2007) showed that the overexpression of type 1 fimbriae dramatically downregulated motility and flagellum expression in *E. coli*. Altogether, these data provided further insight into the complex interplay between type I fimbriae expression and the flagellum-mediated assembly and motility.

These findings demonstrated that *fimA* overexpression could facilitate cell adhesion and contribute to the aggregation effects of *E. coli* during BF formation. However, *fimA* deletion exerted a negative impact on BF formation. Hence, the *fimA* gene can serve as an important regulatory factor for BF formation in *E. coli*.

### Biofilm-based fermentation for L-threonine production

**FIGURE 4:**
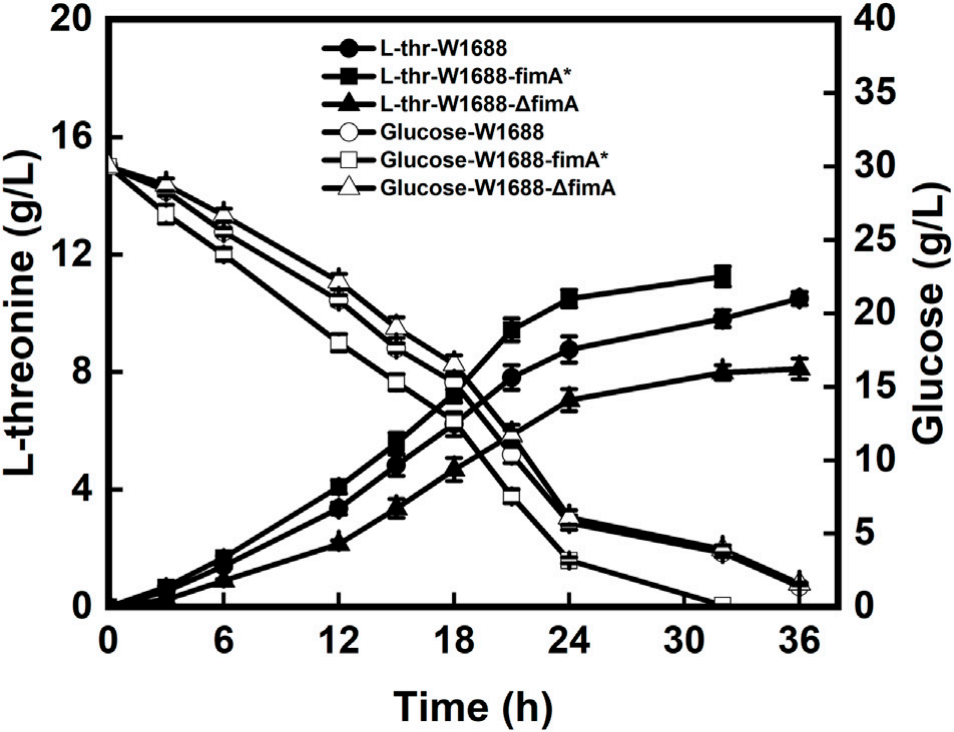
Levels of LT and glucose produced and consumed by the three strains during batch fermentation, respectively.

Batch fermentation of the recombinant and wild-type strains was investigated for the capabilities of BF formation. However, LT production was different from that in *E. coli* W1688-fimH* (Chen et al., 2019). As shown in Figure 4, the LT titer was elevated by 7% in the W1688-fimA* strain compared to the wild-type strain (11.3 g/L versus 10.5 g/L, respectively). In addition, the fermentation cycle was reduced from 36 h to 32 h. Moreover, the level of LT in *E. coli* W1688-ΔfimA was decreased compared to the wild-type strain (8.1 g/L versus 10.5 g/L, respectively) accompanied by the reduced glucose consumption at the first stage of fermentation. Different from the effect of *fimH* on LT production that some enzymes were involved in LT biosynthesis, the significant increase in production for the *fimA* gene had not been observed due to the subtle effect on LT synthesis and metabolism. Instead, the efficiency of BF-based immobilization could be improved, along with the shortened fermentation period of 4 h, thus leading to an increase in BF formation. All these observations indicated that the overexpression of *fimA* might influence the titer and productivity of LT in the *E. coli* W1688-fimA* strain.

A BF-based immobilized fermentation strategy was applied to further improve the efficiency of fermentation. The polyurethane carrier was used to support BFs, which could impose a beneficial effect on cell aggregation. During the immobilized fermentation, the LT titer in the *E. coli* W1688-fimA* strain was almost similar to that in the wild-type strain (data not shown). As a result, BF on the carrier increased significantly, as revealed by SEM observation, and the efficiency of BF immobilization was improved during the continuous LT fermentation of the *E. coli* W1688-fimA* strain. However, the improvement of the LT titer and productivity was still negligible compared to that in the wild-type strain, indicating that the disturbance of *fimA* might be associated with the carbon flux distribution of LT.

**FIGURE 5:**
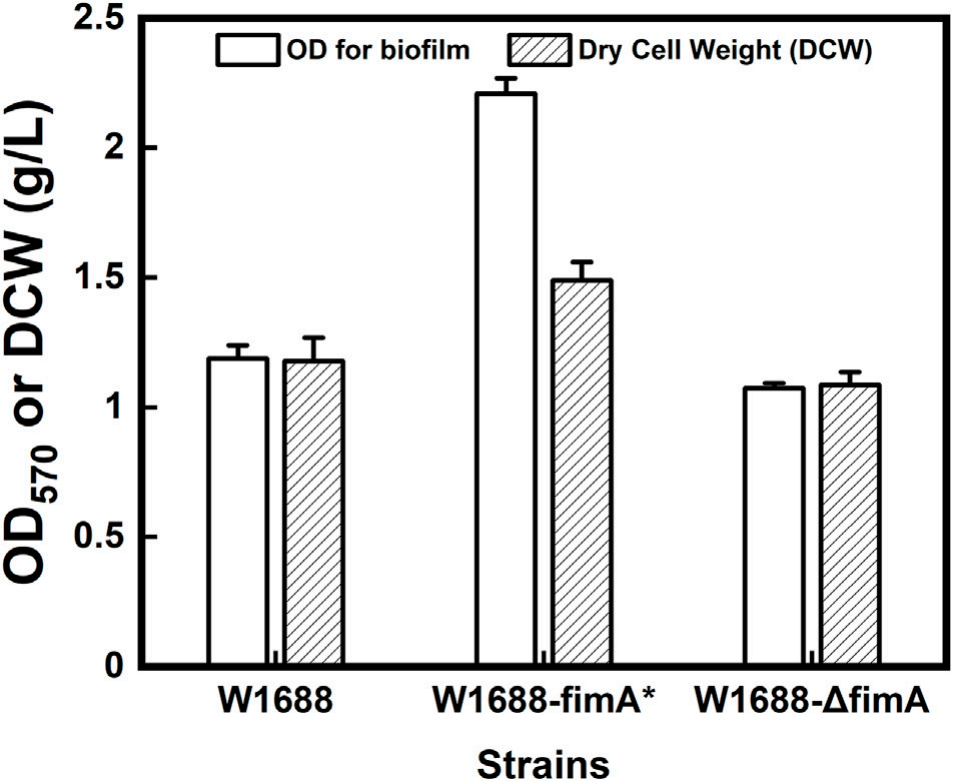
Quantitative analysis of crystal violet staining for BFs and dry cell weight in the three different strains.

To further confirm the carbon flow in the *E. coli* W1688-fimA* strain under the industrial fermentation conditions, dry cell weight (DCW) experiments were performed in the engineered strains. The data on OD_570_ for BF and DCW are shown in Figure 5. The overexpression of the *fimA* gene could lead to enhanced BF formation. In addition, more than 26% of DCW was related to *fimA* overexpression (1.18 g/L versus 1.49 g/L), which was consistent with the tendency of results in OD_570_ for BF formation. Moreover, *fimA* overexpression could lead to the accumulation of extracellular surface fimbriae and filamentous flagella (Figure 3B2).

### Transcriptomic analysis for biofilm formation

**FIGURE 6:**
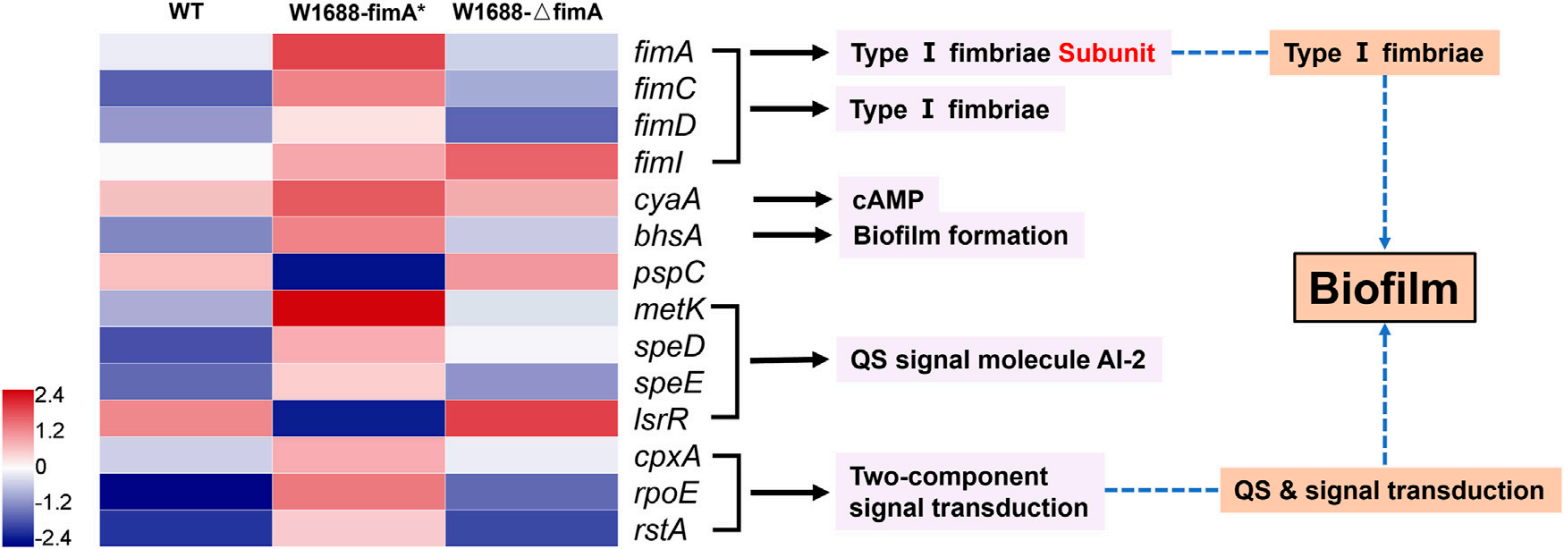
Transcriptomic analysis of genes responsible for the BF formation pathway in the three strains. Blue and red denote downregulated and upregulated genes, respectively.

To explore the mechanisms underlying improved BF formation, transcriptomic analysis was conducted on the recombinant and wild-type strains. The expression ratios of genes related to BF formation were significantly different among the three strains (Figure 6), which could be classified into several distinct clusters. The *E. coli* fimbrial gene cluster is proposed to be composed of a seven-gene operon encoding the structural and assembly components (fimAICDFGH), which shares the same promoter in its 5′ region. The *fimA* gene (encodes for the type Ⅰ fimbriae subunit) and other *fim* family genes guided the assembly of fimbriae to the cell surface (Reisner et al., 2003; Schembri et al., 2003) with high expression in the 1688-fimA* strain. FimI is a putative fimbrial protein and has been shown to encode a 16.4-kDa noncytoplasmic protein product. FimC and FimD are the chaperone and usher proteins used to assemble fimbriae on the surface of the cell, and a number of well-characterized fimbrial systems are assembled using the chaperone/usher pathway (Nuccio and Baumler, 2007). FimC (the “chaperone”) accelerates protein folding and delivers the subunits to the pore-forming protein FimD (“the usher”) in the outer membrane. Subsequently, the subunits are translocated and incorporated into the growing fimbriae (Costello et al., 2012).

In *E. coli*, the cAMP level is primarily regulated by the activity of adenylate cyclase encoded by *cyaA* (Notley-McRobb et al., 1997; Jeong et al., 2021). It catalyzes the production of cAMP from ATP and is a ubiquitous regulatory factor for gene expression and metabolic pathways (Gancedo, 2013; Bassler et al., 2018). BhsA is a stress resistance protein, which is directly related to BF formation under extreme conditions. *metK*, *speD*, and *speE* are associated with the synthesis of quorum-sensing signal molecule autoinducer-2 (AI-2), which in turn can promote BF formation (Vendeville et al., 2005). LsrR is a transcriptional repressor, and its downregulation can accelerate the transport and internalization of AI-2 for more BFs (Schauder et al., 2001; Barrios et al., 2006). In fact, *metK*, which catalyzes the reaction to AI-2, is commonly expressed in prokaryotic bacteria. *cpxA*, *rpoE*, and *rstA* belong to a two-component signal transduction module including signal secretion, reception of the quorum-sensing signal molecule, phosphorylation, and dephosphorylation. They are involved in histidine kinase-mediated cell adhesion, transcriptional control, and gene regulation.

All these genes were modulated to various degrees in the *fimA*-overexpressed and *fimA*-knockout strains compared to the wild-type strain. Our findings indicated that *fimA* overexpression could regulate many other genes to improve BF formation in the fimA* strain.

### Overexpression of *FimA* gene affects L-threonine carbon distribution in *E. coli*

**FIGURE 7:**
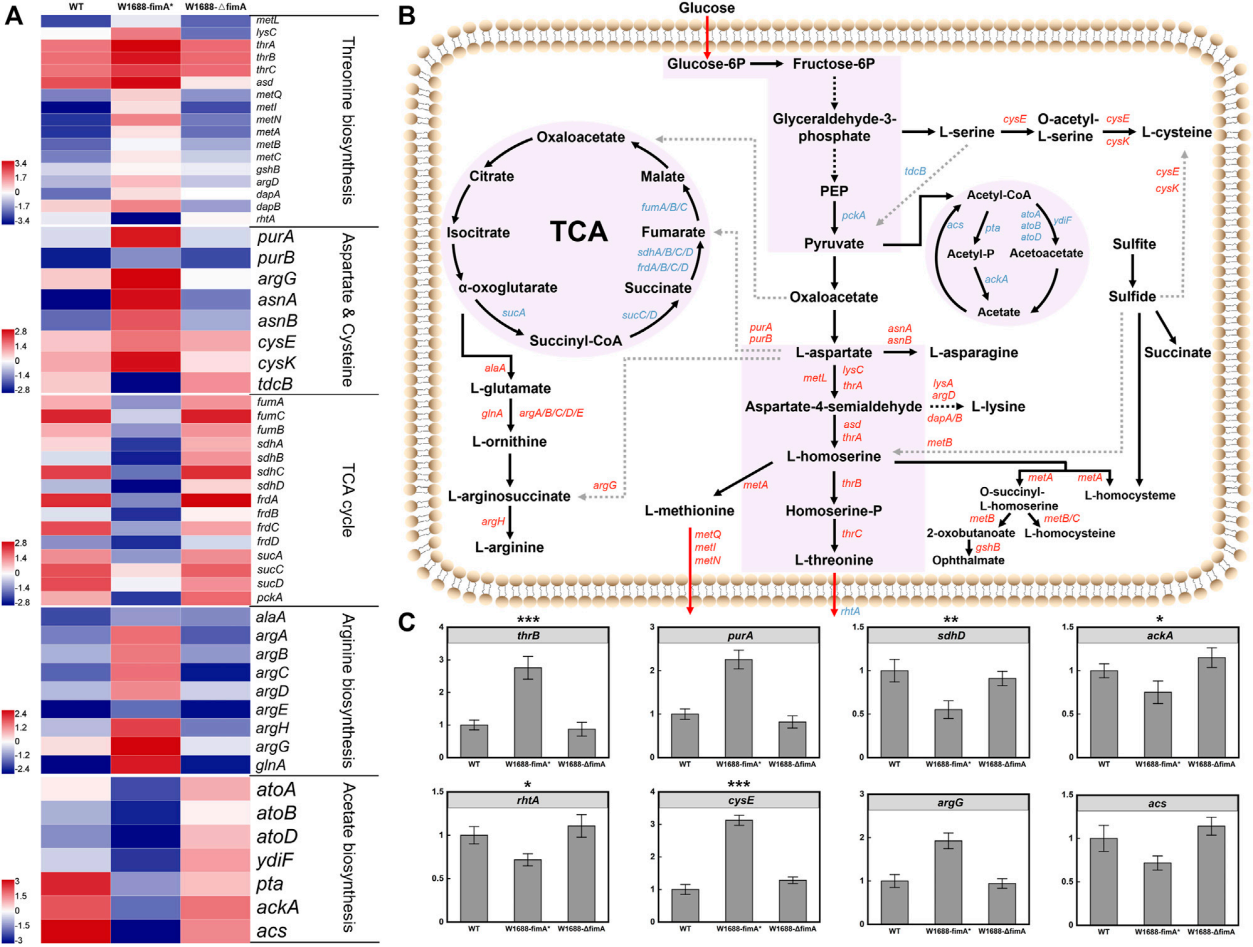
Transcriptomic profiles of LT biosynthetic pathways in three different strains. **(A)** Transcriptomic analysis of the three different strains. Blue and red denote downregulated and upregulated genes, respectively. From the top to the bottom panels: genes in the biosynthesis of LT, L-aspartate, L-cysteine, L-arginine, and acetate biosynthesis and the TCA cycle, respectively; **(B)** LT biosynthetic pathways in *E. coli*. Blue and red arrows indicate downregulated and upregulated genes, respectively. **(C)** RT-qPCR validation of the genes associated with LT biosynthesis and transportation. Mean ± SD (*n* = 3). Two-tailed *t*-test. ***p < 0.001, **p < 0.01, and *p < 0.05.

The overexpression of *fimA* led to the over-assembly of type I fimbriae subunit protein, which was beneficial for cell aggregation. However, BF-based immobilized fermentation was not applicable for increasing LT production. It was preliminarily inferred that *fimA* overexpression could disturb the carbon flow of LT. Hence, transcriptomic analysis was carried out to understand whether the LT titer could regulate biosynthetic pathway genes and clarify the potential mechanisms of carbon distribution in the LT pathway. As expected, *fimA* overexpression not only led to the formation of BFs but also regulated the genes in the LT biosynthetic pathway (Figures 7A,B). For instance, *asd*, *metL*, *lysC,* and *thrA* that convert L-aspartate into L-homoserine, as well as *thrB* and *thrC* that convert L-homoserine into LT (Lee et al., 2003), were all upregulated in *E. coli* W1688-fimA*. In spite of this, the LT transporter-encoding gene *rhtA* was downregulated, thereby decelerating the production of LT (Livshits et al., 2003).

Before the formation of pyruvate, the expression levels of the L-serine and L-cysteine branches were upregulated, which could partly shunt the accumulation of target products. With regard to the L-aspartate branch, *purA/B*, *argG*, and *asnA/B* shared the central carbon metabolism flux of LT, resulting in the accumulation of fumarate, L-arginine, and L-asparagine. Meanwhile, benefiting from the upregulation of *met* family genes, AI-2 biosynthesis was increased, which led to the enhancement of BF formation and L-methionine. The genes of the whole branch from α-oxoglutarate to L-arginine were also upregulated, and they further shunt the carbon flux. In addition, more metabolic precursors of central carbon metabolism could divert to the by-path. Although previous findings have demonstrated that the improved BF formation facilitated the fermentation efficiency of LT, other by-paths also excessively occupied the central carbon flux due to the overexpression of *fimA*.

Indeed, *purA/B* encoding proteins to synthesize target chemical products of the TCA cycle often bring a competitive effect on the carbon flux. However, the weakened TCA cycle may reduce the deoxidization and energy supply to various other biochemical pathways such as NADH and ATP, thus leading to a reduction in the LT titer. Generally, the higher specific growth rates would lead to better production yields. However, it is inevitable that aerobic *E. coli* cultivation will generate acetate, which is caused by an imbalance between the glucose uptake and TCA cycle capacity, resulting in pyruvate and acetyl-CoA accumulation (Lee et al., 2006; Nahku et al., 2010). Transcriptomic analysis showed that the expression levels of *ackA*, *pta*, and *acs* which were about acetate production were noticeably downregulated. Actually, acetate accumulation with the dramatic decrease in pH could reduce the maximum growth rate and inhibit protein production. Meanwhile, it diverts carbon from biomass formation and exhibits harmful effects on BF and cells such as the inhibition of the enzyme activity, changes in the cell membrane permeability and osmotic pressure, and disaggregation of the biofilm. The expression levels of genes in the acetate pathway were downregulated, which might facilitate LT production. Thus, it was confirmed that the glucose consumed by *E. coli* W1688-fimA* in free fermentation was faster than the other strains. The genes in the branched metabolic pathway were differentially expressed, and the genes associated with central carbon metabolism were remarkably upregulated in *E. coli* W1688-fimA* (Figure 7C). Taken altogether, these findings demonstrated that *fimA* overexpression improved the metabolic flux of LT but could also affect other carbon distribution mechanisms.

## Conclusion

In this work, the immobilized fermentation system was proposed for LT production in *E. coli*, together with the benefits of BF formation. The *fimA-*overexpressing strain could promote BF formation under industrial fermentation conditions but is not suitable for continuous immobilized fermentation. The LT titer was only elevated from 10.5 g/L to 11.3 g/L in *E. coli* W1688-fimA*. Transcriptomic profiling indicated that BF formation was improved by modulation of BF-related genes, while LT biosynthesis was affected by genes in the carbon metabolism pathway. In conclusion, the type I fimbriae subunit *fimA* regulated *E. coli* BF formation but affected LT carbon distribution. This study will stimulate thoughts about how the type 1 fimbriae gene regulated cell adhesion and excretion of amino acids and bring some consideration and provide a reference for the development of BF-based fermentation in *E. coli*.

## Data Availability

The datasets presented in this study can be found in online repositories. The names of the repository/repositories and accession number(s) can be found in the article/Supplementary Material. The reads and the HiSeq transcriptomic reads generated for *E. coli* W1688, *E. coli* W1688-fimA* and *E. coli* W1688-ΔfimA, respectively, have been submitted to the BioProject database of National Center for Biotechnology Information (NCBI) under accession number SRR8335002, SRR8335003 and SRR8335000, respectively.
